# NADPH:cytochrome c (P450) reductase activates tirapazamine (SR4233) to restore hypoxic and oxic cytotoxicity in an aerobic resistant derivative of the A549 lung cancer cell line

**DOI:** 10.1054/bjoc.1999.0977

**Published:** 2000-01-18

**Authors:** M P Saunders, A V Patterson, E C Chinje, A L Harris, I J Stratford

**Affiliations:** Department of Pharmacy and Pharmaceutical Sciences, University of Manchester, Oxford Road, Manchester, M13 9PL, UK; ICRF Clinical Oncology Unit, Churchill Hospital, Oxford, OX3 7LJ, UK

**Keywords:** tirapazamine, cytochrome P450 reductase, bioreductive drugs, hypoxia

## Abstract

Tirapazamine (TPZ, SR4233, WIN 59075) is a bioreductive drug that is activated in regions of low oxygen tension to a cytotoxic radical intermediate. This labile metabolite shows high selective toxicity towards hypoxic cells, such as those found in solid tumours. Under aerobic conditions, redox cycling occurs with subsequent generation of superoxide radicals, which are also cytotoxic. NADPH:cytochrome c (P450) reductase (P450R) is a one-electron reducing enzyme that efficiently activates TPZ. Recently a derivative of the A549 non-small cell lung cancer cell line (A549c50) was generated that showed substantially reduced P450R activity compared to its parental line (Elwell et al (1997) *Biochem Pharmacol***54**: 249–257). Here, it is demonstrated that the A549c50 cells are markedly more resistant to TPZ under both aerobic and hypoxic conditions. In addition, these cells have a dramatically impaired ability to metabolize TPZ to its two-electron reduction product, SR4317, under hypoxic conditions when compared to wild-type cells. P450R activity in the A549c50 cells was reintroduced to similar levels as that seen in the parental A549 cells by transfection of the full-length cDNA for human P450R. These P450R over-expressing cells exhibit restored sensitivity to TPZ under both aerobic and hypoxic conditions, comparable to that found in the original parental A549 cells. Further, the ability of the transfected cells to metabolize TPZ to SR4317 under hypoxic conditions is also shown to be restored. This provides further evidence that P450R can play an important role in the activation, metabolism and toxicity of this lead bioreductive drug. © 2000 Cancer Research Campaign


*British Journal of Cancer* (2000) **82**(3), 651–656
© 2000 Cancer Research Campaign
DOI: 10.1054/ bjoc.1999.0977, available online at http://www.idealibrary.com on IDEAL®

### NADPH:cytochrome c (P450) reductase activates tirapazamine (SR4233) to restore hypoxic and oxic cytotoxicity in an aerobic resistant derivative of the A549 lung cancer cell line

**MP Saunders[1], AV Patterson[1], EC Chinje[1], AL Harris[2] and IJ Stratford[1]**

[1]Department of Pharmacy and Pharmaceutical Sciences, University of Manchester, Oxford Road, Manchester, M13 9PL, UK; [2]ICRF Clinical Oncology Unit, Churchill Hospital, Oxford, OX3 7LJ, UK

**Summary** Tirapazamine (TPZ, SR4233, WIN 59075) is a bioreductive drug that is activated in regions of low oxygen tension to a cytotoxic radical intermediate. This labile metabolite shows high selective toxicity towards hypoxic cells, such as those found in solid tumours. Under aerobic conditions, redox cycling occurs with subsequent generation of superoxide radicals, which are also cytotoxic. NADPH:cytochrome c (P450) reductase (P450R) is a one-electron reducing enzyme that efficiently activates TPZ. Recently a derivative of the A549 non-small cell lung cancer cell line (A549c50) was generated that showed substantially reduced P450R activity compared to its parental line (Elwell et al (1997) *Biochem Pharmacol* **54:** 249–257). Here, it is demonstrated that the A549c50 cells are markedly more resistant to TPZ under both aerobic and hypoxic conditions. In addition, these cells have a dramatically impaired ability to metabolize TPZ to its two-electron reduction product, SR4317, under hypoxic conditions when compared to wild-type cells. P450R activity in the A549c50 cells was reintroduced to similar levels as that seen in the parental A549 cells by transfection of the full-length cDNA for human P450R. These P450R over-expressing cells exhibit restored sensitivity to TPZ under both aerobic and hypoxic conditions, comparable to that found in the original parental A549 cells. Further, the ability of the transfected cells to metabolize TPZ to SR4317 under hypoxic conditions is also shown to be restored. This provides further evidence that P450R can play an important role in the activation, metabolism and toxicity of this lead bioreductive drug. © 2000 Cancer Research Campaign

**Keywords:** tirapazamine; cytochrome P450 reductase; bioreductive drugs; hypoxia

Tumours have a disorganized vascular supply creating foci of low oxygen tension (Chaplin et al, 1987; Jain, 1988). Hypoxic cells in these regions are resistant to treatment with radiotherapy, predisposing to failure of this modality of treatment. Consequently, attempts to overcome tumour hypoxia have been made with a certain degree of success (Overgaard, 1992). Since normal tissues are usually oxic in comparison with tumours (Vaupel, 1993), this difference in oxygenation can be exploited for beneficial effect. One way of achieving this is to use bioreductive drugs which selectively kill hypoxic cells (Workman and Stratford, 1993; Adams and Stratford, 1994). These prodrugs require metabolic activation by enzymes including the one-electron dontor, NADPH:cytochrome c (P450) reductase (P450R). Endogenous P450 reductase activity in tumours can be heterogeneous (Forrester et al, 1994; Forkert et al, 1996). To overcome this, it has been proposed that the introduction of P450 reductase into tumours as a therapeutic gene may allow more effective targeting of the hypoxic areas in tumours by bioreductive drugs (Patterson et al, 1997).

Tirapazamine (TPZ, SR4233) is a bioreductive drug that is activated by one-electron reduction to a cytotoxic radical intermediate prior to further reduction to the non-toxic metabolite SR4317. The TPZ radical has been shown to elicit selective toxicity towards hypoxic cells, because under aerobic conditions it is quenched by molecular oxygen (Brown, 1993). Redox cycling, with the generation of superoxide radicals is believed to be the cause of underlying oxic cytotoxicity (Lloyd et al, 1991). Because of the selective toxicity of TPZ towards hypoxic cells, preclinical studies concentrated on combining this bioreductive cytotoxin with either radiation (Brown and Lemmon, 1990) or other chemotherapeutic drugs such as cisplatin (Dorie and Brown, 1993). These promising preclinical studies have resulted in TPZ undergoing clinical evaluation (Rodriguez et al, 1996; Lee et al, 1998) and it is currently in phase II and III clinical trials as an adjunct to cisplatin-based chemotherapy or radiation.

Elwell et al (1997) evaluated the biochemical consequences of chronic aerobic tirapazamine exposure in an effort to determine the mechanism of oxic toxicity. The A549 wild type (wt) cell line was grown continuously in incremental concentrations of TPZ (10, 25, 50 and 100 μM) over a period of approximately 6 months. Ultimately, the derived lines maintained aerobic resistance to TPZ without any further selective pressure. The selected cell lineages were found to have significantly elevated levels of manganese superoxide dismutase (MnSOD), along with considerably reduced DT-diaphorase and P450R activity (9.4-, 35.6- and 78.6-fold respectively). The selected lines were much more resistant to TPZ under aerobic conditions (using colony survival assays). This oxic resistance was attributed to both the elevated levels of MnSOD

*Received 12 March 1999*
*Revised 7 August 1999*
*Accepted 9 August 1999*

*Correspondence to:* IJ Stratford and the reduced P450R activity. It was reasoned that since MnSOD is an effective radical scavenger it would scavenge any oxygen radical byproducts generated through redox-cycling, and so moderate the aerobic toxicity of this drug (Elwell et al, 1997). As a consequence of the greatly reduced P450R activity, the selected cells might have been predicted to be more resistant to TPZ under both aerobic and hypoxic conditions. However, this was apparently not the case, with the selected lines being only slightly more resistant under hypoxic conditions (1.5-fold). Additionally, hypoxic TPZ metabolism remained intact and did not alter significantly. Considered together, these results suggested that P450R was involved in the aerobic toxicity of TPZ, but was not an important factor in the hypoxic activation of this drug. In contrast, there is another body of evidence showing that P450R does play a dominant role in the reductive activation, toxicity and metabolism of TPZ. For example, in a study of six breast cancer cell lines, it was shown that cellular P450R activity correlated strongly with both hypoxic metabolism and toxicity of TPZ (Patterson et al, 1995). Subsequently the breast cancer cell, MDA231, was transfected with an expression vector containing human P450R. Stably transfected clones were selected that showed a 50-fold range in P450R activity and using these clones the importance of P450R was unambiguously demonstrated for both hypoxic toxicity and metabolism of TPZ (Patterson et al, 1997). Thus, there are two apparently opposing interpretations regarding the importance of P450R for TPZ activation and toxicity under hypoxic conditions. Therefore, in this paper, we have taken the A549c50 cells that show deficient P450R activity (Elwell et al, 1997) and transfected them with the cDNA encoding P450R. Stably transfected clones were selected and compared for TPZ toxicity and metabolism with both the parental A549 cells and the A549c50 cells derived from them.

### MATERIALS AND METHODS

### Chemicals

SR4233 was made by a method described by Seng and Ley (1972) and was synthesized in the Medicinal Chemistry Section of the School of Pharmacy, Manchester University. NADPH and NADH were purchased from Boehringer Mannheim (Lewes, UK). Tissue culture media (Dulbecc's modified Eagle's medium, DMEM) was obtained from Gibco-BRL. Fetal calf serum (FCS) and all other reagents of analytical grade were purchased from Sigma (Poole, UK).

### Cells and culture

A549 wt and A549c50 cells (Elwell et al, 1997) were grown in DMEM medium supplemented with 2 mM glutamine and 10% FCS. The clones were grown in the same media although it also contained puromycin 2 μg $ml^{-1}$. All cell lines were maintained in an exponential growth phase and only clones of five or fewer passages from their initial selection were used in this project.

### Transfection and clonal selection

Details of the expression vector for human P450R cDNA have been described previously (Patterson et al, 1997). A549c50 cells in exponential growth phase were harvested and suspended in 'Optimix' buffer (Equibio) to increase cell survival after transfection. Approximately $1 \times 10^7$ cells were mixed with 20 μg of pBABE–puro–reductase vector. After 2 min of equilibration, the sample was electroporated (1500 μF, 290 V) and then immediately returned to a 15 cm plate containing pre-warmed media. Forty-eight hours after transfection, puromycin containing media (2 μg $ml^{-1}$) was added to the plate. Eighteen days after transfection, 48 colonies were picked and seeded into individual wells of a 24-well plate. The cells were maintained under puromycin selection at all times.

### Tumour cell lysates

Cells in exponential growth phase were washed twice with phosphate-buffered saline (PBS) and then scraped in ice-cold PBS. After centrifugation at 100 ***g*** for 10 min at 4°C, the PBS was discarded and the pellet resuspended in 200–500 μl of ice-cold nuclear buffer A (10 mM HEPES, 1.5 mM magnesium chloride, 10 mM potassium chloride, 0.05 mM dithiothreitol, pH 7.4). After standing for 10 min in ice, the suspension was sonicated using an MSE soniprep 150 ($3 \times 5$ s, 23 kHz, oscillation amplitude ~ 10 μm). Samples were placed on ice between each sonication. After standing for a further 10 min at 4°C, the sample was centrifuged at 7800 ***g*** for 15 min at 4°C. The resulting lysate was snap-frozen and then stored at –70°C. The protein concentration was determined using the Pierce assay with bovine serum albumin (BSA) as the standard for comparison.

### Clonal characterization

Cell lysates made from the A549wt, A549c50 and clonal cell lines, were assayed for P450R, DT-diaphorase and TPZ metabolism. The mean from at least three different lysates was taken as the final result.

P450 reductase activity was determined spectrophotometrically as the NADPH-dependent reduction of cytochrome c (Patterson et al, 1997). Each incubation comprised 400 μl of cytochrome c (final concentration 50 μM), 100 μl of 10 mM potassium cyanide (final concentration 1 mM) and 10–300 μl of lysate made up to 0.98 ml with 100 mM phosphate buffer, pH 7.6. The reaction was equilibrated at 37°C and was initiated by the addition of 20 μl of 10 mM NADPH (final concentration 200 μM). The rate of reduction of cytochrome c was monitored at 550 nm for 2 min against a blank without NADPH. Initial rates of reaction were based on an extinction coefficient of 21 $mM^{-1}$ $cm^{-1}$ calculated and expressed as nmol cytochrome c reduced per min per mg of lysate protein.

DT-diaphorase was determined spectrophotometrically as the dicoumorol-inhibitable component of the NADPH-dependent reduction of cytochrome c (Robertson et al, 1994). Activity was again expressed as nmol cytochrome c reduced per min per mg of lysate protein.

### Metabolism of tirapazamine by cell lysates

Fifty microlitres of lysate was incubated at 37°C under nitrogen gassing in the presence of excess NADPH (100 μl of 5 mM) and buffer (0.2 M potassium phosphate pH 7.4). The final volume was 500 μl. After 10 min, 20 μl of 20 mM SR4233 in dimethyl sulphoxide (DMSO) was added to start the reaction. Forty minutes later the reaction was stopped by pipetting 200 μl of this reaction

© 2000 Cancer Research Campaign mixture to a methanol–buffer solution. An internal standard (4-nitroquinoline) was also added to the tube. The supernatants were transferred to autosampler vials for analysis by isocratic reverse-phase high-performance liquid chromatography (HPLC) (Patterson et al, 1997). The amount of each component was detected spectrophotometrically at 267 nm. The values were then corrected for protein content and expressed as nmol SR4317 formed per min per mg protein.

### Drug sensitivity

### *MTT proliferation assay*

Details of this assay have been described (Patterson et al, 1995). Prior to the drug sensitivity studies the seeding density required to give an optimum optical density following metabolism of 3-(4,5-dimethylthiazal-2-yl)-2,5-diphenyltetrazolium bromide (MTT), was determined by growing increasing numbers (1000–10 000 cells per well) of the A549 and derived cell lines in a 96-well plate for 4 days. At this point the plate was assayed and a seeding density was chosen that gave an optical density of approximately 0.5 units (Labtech Anthos ht III 96-well plate spectrometer). The A549c50 cells and transfected clonal lines grew at similar rates and were seeded at 7000 cells per well. The A549wt cells grew more rapidly and were seeded at 4000 cells per well.

TPZ was dissolved in DMSO and stored at –70°C (40 mM stock). A fresh aliquot was used for every experiment. TPZ was diluted in fresh media to make nine concentrations to be added to the remaining columns of the 96-well plate.

Cells in exponential growth phase were washed twice in sterile PBS and then harvested from the flask. After counting, cells were seeded into 11 out of 12 columns of a 96-well plate. The first column contained only media. After allowing the cells to adhere for 1 h, they were exposed to increasing concentrations of TPZ. The second and third columns contained only cells in media and were used as controls. Cells were exposed to the drug for 3 h under aerobic or hypoxic conditions. All hypoxic exposures were conducted under conditions where residual oxygen in the gas phase was removed by passage over palladium catalyst. All plastics and media were preincubated in hypoxia for at least 24 h to remove any residual oxygen, prior to use. After each 3-h exposure, the drug was removed and fresh media instilled into each well. Plates were then incubated for 96 h in air at 37°C, prior to determining cell number using the MTT assay. The concentration of drug required to reduce the optical density to 50% of that of the control, $IC_{50}$, was used as a measure of toxicity of the particular drug treatment in a given cell line.

### *Clonogenic survival assay*

Cells in exponential growth phase were washed twice in PBS, harvested, counted and seeded at different densities under hypoxic conditions into 6-well plates. Seeding density depended on the severity of the subsequent treatment and ranged from $10^2$ to $10^7$ cells per well. The cells were allowed to adhere for 1 h before they were exposed to TPZ for 3 h under catalyst-induced hypoxia. The drug was removed and fresh media added to the wells. The plates were then incubated at 37°C for between 2 and 4 weeks. For every individual clonogenic survival determination, three to four duplicate wells were exposed to each concentration of TPZ. Experiments were repeated four times.

When colonies were seen in the control wells (no drug), the media was discarded and the cells fixed for 1 min with 70% alcohol. Bromophenol blue (0.25%) in 20% ethanol was then added to the wells for 15 min. This was then discarded and the plate carefully washed in tap water. After drying, the number of mauve-stained colonies (> 50 cells) in each well were counted.

**Table 1** A549 wt, A549c50 and P450R transfected cell lines. Determination of P450R, DT-diaphorase activity and SR4317 formation ($IC_{50}$ ± s.d., number of assays shown in brackets)

| Cell line | P450R (nmol cyt c $min^{-1}$ $mg^{-1}$) | DT-diaphorase (nmol cyt c $min^{-1}$ $mg^{-1}$) | SR4317 formation (nmol $min^{-1}$ $mg^{-1}$ protein) |
|---|---|---|---|
| A549c50 | 0.76 ± 0.15 (5) | 361 ± 67 (5) | 2.62 + 0.42 (5) |
| Clone 10 | 7.09 ± 1.99 (8) | 403 ± 44 (5) | 11.92 + 2.31 (5) |
| A549 wt | 22.79 ± 3.84 (7) | 1641 ± 325 (7) | 15.94 + 2.51 (5) |
| Clone 46 | 34.76 ± 1.70 (5) | 269 ± 97 (4) | 29.11 + 2.69 (5) |

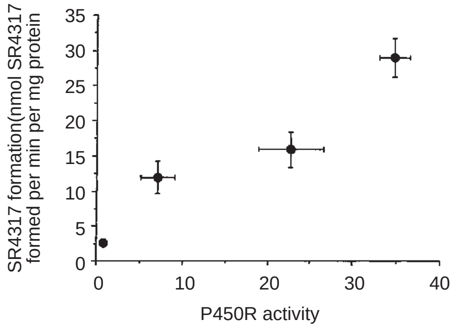

**Figure 1** Dependence on the expression of P450R, measured as nmol cyt c reduced per min per mg cell lysate protein, in the A549 wt, A549 c50 and transfected clones, for hypoxic metabolism of TPZ to SR 4317. (Error bars = standard deviations)

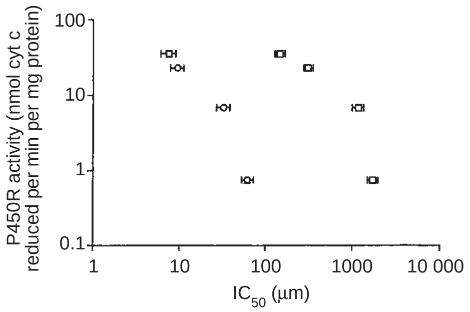

**Figure 2** Dependence of the $IC_{50}$ of TPZ in the A549 wt and transfected clones, on the activity of P450R in the cells. Drug exposures were for 23 h in air (□) or hypoxia (○). Values of $IC_{50}$ were determined using the MTT assay. (Error bars = standard errors from at least 3 individual experiments)

### Statistical analysis

The data was analysed by methods previously described (Patterson et al, 1995). Statistical significance was tested by the Student's *t*-test.

*© 2000 Cancer Research Campaign*

**Table 2** Response to TPZ under aerobic and hypoxic conditions ($IC_{50}$ ± s.d., number of exposures shown in brackets

| Cell line | 3-hour aerobic | 3-hour hypoxic | Differential toxicity |
|---|---|---|---|
| A549c50 | 1785 ± 528 (4) | 63.0 ± 19.8 (6) | 28.3 |
| Clone 10 | 1185 ± 188 (3) | 33.0 ± 10.3 (5) | 35.9 |
| A549 wt | 323 ± 83 (5) | 11.0 ± 7.2 (6) | 29.4 |
| Clone 46 | 148 ± 27 (4) | 7.4 ± 1.0 (4) | 20.0 |

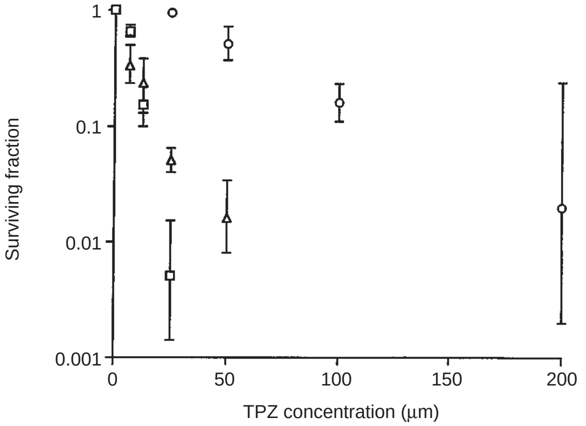

**Figure 3** Cells exposed to TPZ for 3 hours under hypoxic conditions. Dependence of surviving fraction, as assayed using clonogenic survival, on TPZ concentration. A459 wt (□): A549c50 (○); clone 46 (Δ). (Error bars = standard deviations)

### RESULTS

P450 reductase (P450R) activity was measured in A549 wt, A549c50, and two isolated clonal cell lines (clone 10 and clone 46) that displayed P450R activities above and below that of the parental A549 cell line. The results are displayed in Table 1. Elevation in P450R activity above the A549c50 cell line ranged from 9.3-fold in clone 10 to 45.7 times in the highest expressing clone (clone 46). The P450R activity for the A549wt cell line was found to be midway between these two values (30 times greater than the A549c50 cell line).

The A549c50 cell line had fivefold lower DT-diaphorase activity than the A549wt cell line (Elwell et al, 1997). We have confirmed this observation (Table 1) and to ensure that the transfection of P450R did not modify the activity of this enzyme, potentially important in the activation of TPZ, we have also carried out measurements in the clonal lines 10 and 46. Values of activity are given in Table 1 and indicate that both of the clones had similar DT-diaphorase activity to the A549c50 cell line.

To determine whether overexpression of P450R conferred elevated metabolism of TPZ in the clonal lines, the rate at which cell lysates reduced TPZ to its two-electron reduction product (SR4317) under hypoxic condition was determined. Data plotted in Figure 1 shows that there is a good correlation between P450R activity and SR4317 formation and that this was highly significant (slope = 1.69 ± 0.22; $P = 0.017$).

The cytotoxicity of TPZ under aerobic and hypoxic conditions was determined using the MTT proliferation assay. Values of $IC_{50}$ and a summary of these results is tabulated in Table 2 and displayed graphically in Figure 2. There was a clear relationship between P450R activity of cell lysate and TPZ cytotoxicity under both aerobic and hypoxic conditions. In hypoxia, slope = –1.66 ± 0.29; $P = 0.0023$. The greatest toxicity (lowest $IC_{50}$), occurred in clone 46, which had the highest P450R activity, whilst the value for the A549wt cell line fell between that of the two clones. The results for the clonogenic survival of the different cell types exposed to tirapazamine under hypoxic conditions are given in Figure 3. The control plating efficiency for the cell lines was:A549wt, 22.50% ± 8.06 ($n = 4$); A549c50, 3.06% ± 2.00 ($n = 4$); Clone 46, 3.74% ± 2.14 ($n = 4$). From the data it can be seen that the transfection of P450R into the A549c50 cell line restores the hypoxic sensitivity of clone 46 to tirapazamine. These data are therefore consistent with the results obtained using the MTT proliferation assay.

### DISCUSSION

We have successfully introduced an expression cassette encoding the full length cDNA for human P450R into a derivative of the A549 NSCLC cell line (A549c50), which had low endogenous levels of this flavoenzyme. Two representative clones were selected which had P450R activity above and below that of the parental cell line. Under both aerobic and hypoxic conditions, P450R was found to be strongly implicated in the activation and subsequent toxicity of TPZ. This was consistent with the previous findings in both a panel of breast cancer cell lines (Patterson et al, 1995) and P450R over-expressing MDA231 clones (Patterson et al, 1997).

By restoring P450R activity to the A549c50 cell line, the sensitivity to tirapazamine, as determined by the MTT proliferation assay, increased under both aerobic and hypoxic conditions (Figure 2). To further evaluate the role of P450R in the hypoxic sensitivity to tirapazamine, clonogenic survival assays were performed. Clone 46, which had a P450R activity slightly above that of the parental line, was compared to the latter and to the A549c50 cell line from which it was derived. We found that the hypoxic sensitivity was fully restored by the introduction of P450R into the resistant A549c50 cell line (Figure 3).

It is of note that clone 46 has moderately greater P450R activity compared to the A549 wt cell line (35 v 23 nmol cyt c reduced $min^{-1}$ $mg^{-1}$), and so might be anticipated to exhibit hypersensitivity to TPZ. Yet a number of other metabolic differences existed between clone 46 and the A549 wt cell line. These included reduced DT-diaphorase activity (Table 1) and elevated MnSOD levels which were found in the parental A549c50 cell line. Riley et al (1993) have shown that murine DTD is not significantly involved in the anaerobic microsomal reduction of TPZ. Further, Patterson et al (1994) did not observe a relationship between DTD activity and either aerobic or hypoxic TPZ sensitivity in a panel of 18 human lung and breast carcinoma cell lines. The apparent inability of the 5000-fold range in DTD activity to influence the 12-fold range in TPZ sensitivity in vitro, together with the lack of NAD(P)H-dependent SR4317 formation under aerobic conditions, strongly argues against a significant role for human DTD in the metabolism and cytotoxicity of TPZ. This is confirmed in studies by Plumb and Workman (1994) who used two colon carcinoma

© 2000 Cancer Research Campaign cell lines, HT29 and BE which have large differences in DT diaphorase activity but essentially similar sensitivities to TPZ. In contrast the difference in MnSOD levels may explain the relative resistance of this clone in air, since the excess MnSOD would scavenge the superoxide radicals so reducing the sensitivity to TPZ. Also, since many changes were induced by chronic TPZ exposure, it would not be unreasonable to suggest that other unknown differences also exist, that might contribute to the relative aerobic resistance of clone 46.

It is thought that the mechanisms of aerobic and hypoxic cytotoxicity are distinct (Brown, 1993). Under hypoxic conditions a free radical intermediate is generated by one electron reduction of the parent compound (Lloyd et al, 1991). This cytotoxic radical is believed to abstract a hydrogen atom from DNA and produce multiple single and double strand-breaks which ultimately lead to chromosomal defects. Under aerobic conditions, the unstable tirapazamine radical is rapidly back-oxidized to the parent compound, with the subsequent generation of superoxide radicals, which are in themselves moderately cytotoxic (Lloyd et al, 1991). Thus, although the mechanisms of toxicity are distinct, P450R is implicated in both the aerobic and hypoxic activation of tirapazamine. The current results, together with those of Patterson et al (1997), establish unequivocally that transfection of the cDNA for P450 reductase into human tumour cells increases both the aerobic and hypoxic sensitivity to tirapazamine. The data published by Elwell et al (1997) showed that the derived cell lines with reduced P450R activity were much more resistant to TPZ under aerobic conditions but were only minimally less sensitive under hypoxic conditions. This selective loss of aerobic but not hypoxic toxicity reported by Elwell et al (1997) is difficult to reconcile considering the known redox properties of P450R. Since this flavoenzyme exists as an air-stable semiquinone radical, probably FAD-FMNH (Vermilion and Coon, 1978; Yasukochi et al, 1979), it will donate electrons to any appropiate artificial electron acceptor (i.e. TPZ). The presence or absence of molecular oxygen will not markedly influence its catalytic properties. The hypoxia selectivity is a distinct property of the TPZ radical species and its interrelationship with the oxygen couple. By implication this is independent of its mechanism of activation.

SR4317 formation and colony survival results obtained here differ from those obtained by Elwell et al (1997) with the same cell lines. Elwell et al (1997) did not find a significant difference in either SR4317 formation or hypoxic sensitivity to tirapazamine, between the A549wt and A549c50 cell lines. It is possible that these discrepancies may reflect the different techniques used. To determine SR4317 formation, Elwell et al (1997) used whole cells whilst in this report metabolism was determined using S-9 fractions derived from cell lysates in the presence of excess amounts of NADPH as a cofactor. It is possible that the availability of NADPH as a cofactor in drug activation may well be influenced by prevailing metabolic conditions, forming a coupled interactive system and therefore constituting a control point in drug activation. The data obtained based on the methodology used by Elwell and colleagues (Elwell et al, 1997) indicated that SR4317 formation rate was essentially identical between the A549wt and A549c50 cells despite the very dramatic differences in P450R activity. On the contrary the data reported here clearly establishes that the differences seen in P450R activity are also reflected in the relative amplitude of SR4317 formation rates. This is consistent with the well documented contention of a major role played by P450R in TPZ metabolism under hypoxia. Further, in our experiments, the colony survival results agree with the MTT proliferation assay results and both sets of data are also compatible with the hypothesis that P450R is involved with the hypoxic activation of TPZ.

The contribution of other intracellular reductases in the activation of TPZ has been examined and is still unclear (Patterson et al, 1998). Both P450R and the cytochrome P450 family are capable of metabolizing TPZ. The contention that P450R may not play a dominant role in TPZ cytotoxicity was put forward by Evans et al (1998) who suggested that only radicals generated in the nucleus would contribute to intranuclear DNA damage. Since P450R was not detected in nuclear fractions, consistent with its microsomal localization, it was eliminated as the candidate intranuclear enzyme responsible for TPZ metabolism. Yet, since the reticular membrane is closely associated with the nuclear membrane and the TPZ radical is thought to be sufficiently stable to diffuse over significant distances (Laderoute et al, 1988), it seems reasonable that cytotoxic radicals generated by reduction of TPZ in the reticular membrane may be able to diffuse into the nucleus. Irrespective of the site of radical generation, our results show that elevated expression of P450R makes human tumour cells more sensitive to TPZ under both aerobic and hypoxic conditions.

Hypoxia can be considered a tumour phenomenon since the oxygen tension is often low compared to that of the adjacent normal tissues. Bioreductive drugs take advantage of this physiological difference by eliciting selective toxicity towards hypoxic cells. We have shown that transfection of the cDNA for P450R into a NSCLC cell line enhances the aerobic and hypoxic cytotoxicity of TPZ. This is in direct agreement with previous work published by Patterson et al (1997) using a breast cancer cell line. In the same study, high P450R expressing clones were also more sensitive to the 2-nitroimidazole, RSU1069, under hypoxic conditions. Using identical clones, a similar result was obtained with indolequinone EO9 and two related analogues (Saunders et al, 1996). In summary, we have shown that transfection of the full-length cDNA for P450R into both breast cancer and non-small-cell lung cancer cell lines increases the hypoxic sensitivity to TPZ and also a wide range of other bioreductive drugs.

### ACKNOWLEDGEMENTS

This work was funded in part by the Medical Research Council (MRC) and AICR. MPS is a MRC clinical training fellow. Statistical analysis was conducted by Mr D Papworth. We are grateful to Professor CR Wolf for providing the P450R cDNA. We thank Dr M Jaffar for synthesizing the TPZ used in this study. The A549c50 cell line was kindly provided by JM Brown, Division of Radiation Biology, Department of Radiation Oncology, Stanford University, Stanford, CA, USA.

### REFERENCES

Adams GE and Stratford IJ (1994) Bioreductive drugs for cancer therapy: the search for tumor specificity. *Int J Radiat Oncol Biol Phys* **29**: 231–238

Brown JM (1993) SR4233 (Tirapazamine): a new anticancer drug exploiting hypoxia in solid tumours. *Br J Cancer* **67**: 1163–1170

Brown JM and Lemmon MJ (1990) Potentiation by the hypoxic cytotoxin SR4233 of cell killing produced by fractionated irradiation of mouse tumours. *Cancer Res* **50**: 7745–7749

Chaplin DJ, Olive PL and Durrand RE (1987) Intermittent blood flow in a murine tumour: radiobiological effects. *Cancer Res* **47**: 597–601

© 2000 Cancer Research Campaign 

Dorie MJ and Brown JM (1993) Tumour-specific schedule dependent interaction between tirapazamine (SR 4233) and cisplatin. *Cancer Res* **53**: 4633–4636

Elwell JH, Siim BG, Evans JW and Brown JM (1997) Adaption of human tumour cells to tirapazamine under aerobic conditions. *Biochem Pharmacol* **54**: 249–257

Evans JW, Yudoh K, Delahoussaye YM and Brown JM (1998) Tirapazamine is metabolised to its DNA-damaging radical by intranuclear enzymes. *Cancer Res* **58**: 2098–2101

Forkert PG, Lord JA and Parkinson A (1996) Alterations in expression of CYP1A1 and NADPH-cytochrome P450 reductase during lung tumor development in SWR/J mice. *Carcinogenesis* **17**: 127–134

Forrester LM, Hayes JD, Millis R, Barnes D, Harris AL, Schlager JJ, Powis G and Wolf CR (1990) Expression of glutathione S-transferases and cytochrome P450 in normal and tumor breast tissue. *Carcinogenesis* **11**: 2163–2170

Jain RK (1988) Determinants of tumour blood flow. *Cancer Res* **48**: 2641–2658

Laderoute K, Wardman P and Rauth AM (1988) Molecular mechanisms for the hypoxic-dependent activation of 3-amino-1,2,4-benzotriazene-1,4-dioxide (SR4233). *Biochem Pharmacol* **37**: 1487–1495

Lee D, Trotti A, Spencer S, Rostock R, Fisher C, von Roemeling R, Harvey E and Groves E (1998) A phase II trial of radiotherapy with concurrent tirapazamine, a hypoxic cytotoxin, for advanced head and neck carcinomas. *Int J of Radiat Oncol Biol Phys* (in press)

Lloyd RV, Duling DR, Rumyantseva GV, Mason RP and Bridson PK (1991) Microsomal reduction of 3-amino-1,2,4-benzotriazine 1,4-dioxide to a free radical. *Mol Pharmacol* 40: 440–445

Overgaard J (1992) Importance of tumour hypoxia in radiotherapy: a meta-analysis of controlled clinical trials. *Radiother Oncol* **24**: Abs-S64

Patterson AV, Robertson N, Houlbrook S, Stephens MA, Adams GE, Harris AL, Stratford IJ and Carmichael J (1994) The role of DT-diaphorase in determining the sensitivity of human tumour cell to tirapazamine (SR 4233). *Int J Radiat Oncol Biol Phys* **29**: 369

Patterson AV, Barham HM, Chinje EC, Adams, GE, Harris AL and Stratford IJ (1995) Importance of P450 reductase activity in determining sensitivity of breast tumour cells to the bioreductive drug, tirapazamine (SR 4233). *Br J Cancer* **72**: 1144–1150

Patterson AV, Saunders MP, Chinje EC, Talbot DC, Harris AL and Stratford IJ (1997) Overexpression of human NADPH:cytochrome c (P450) reductase confers enhanced sensitivity to both tirapazamine (SR4233) and RSU1069. *Br J Cancer* **76**: 1338–1347

Patterson AV, Saunders MP, Chinje EC, Patterson LH and Stratford IJ (1998) Enzymology of tirapazamine metabolism: a review. *Anti-Cancer Drug Design* **13**: 541–573

Plumb JA and Workman P (1994) Unusually marked hypoxic sensitization to indoloquinone EO9 and mitomycin C in a human colon-tumour cell line that lacks DT-diaphorase activity. *Int J Cancer* **56**: 134

Riley JR, Hemingway SA, Graham MA, Workman P (1993) Initial characterisation of the major mouse cytochrome P450 enzymes involved in the reductive metabolism of the hypoxic cytotoxin 3-amino-1,2,4-benzotrizene-1,4-di-*N*-oxide (Tirapazamine, SR 4233, WIN59075). *Biochem Pharmacol* **45**: 1065

Robertson N, Haigh A, Adams GE and Stratford IJ (1994) Factors affecting sensitivity to EO9 in rodent and human tumour cells in vitro: DT-diaphorase activity and hypoxia. *Eur J Cancer* 30A: 1013–1019

Rodriguez GI, Valdivieso M, Von Hoff DD, Kraut M, Burris HA, Eckardt JR, Lockwood G, Kennedy H and von Roemeling R (1996) A phase I/II trial of the combination of tirapazamine and cisplatin in patients with non-small cell lung cancer (NSCLC) (Meeting abstract). *Proc Annu Meet Am Soc Clin Oncol* 15: 382

Saunders MP, Patterson AV, Jaffar M, Harris AL and Stratford IJ (1996) Structural requirements for EO9 toxicity and dependence on P450 reductase for activation. *Br J Cancer* supp 73: XXVI, Abs-P6

Vaupel P (1993) Oxygenation of solid tumours. In: *Drug Resistance in Oncology*, Teicher BA (ed), pp. 59–85. Marcel Dekker: New York

Workman P and Stratford IJ (1993) The experimental development of bioreductive drugs and their role in cancer therapy. *Cancer Metastasis Rev* 12: 73–82

*© 2000 Cancer Research Campaign*

## References

[PDF_00531] Adams G. E., Stratford I. J. (1994). Bioreductive drugs for cancer therapy: the search for tumor specificity.. Int J Radiat Oncol Biol Phys.

[PDF_00535] Brown J. M., Lemmon M. J. (1990). Potentiation by the hypoxic cytotoxin SR 4233 of cell killing produced by fractionated irradiation of mouse tumors.. Cancer Res.

[PDF_00533] Brown J. M. (1993). SR 4233 (tirapazamine): a new anticancer drug exploiting hypoxia in solid tumours.. Br J Cancer.

[PDF_00538] Chaplin D. J., Olive P. L., Durand R. E. (1987). Intermittent blood flow in a murine tumor: radiobiological effects.. Cancer Res.

[PDF_00542] Dorie M. J., Brown J. M. (1993). Tumor-specific, schedule-dependent interaction between tirapazamine (SR 4233) and cisplatin.. Cancer Res.

[PDF_00545] Elwell J. H., Siim B. G., Evans J. W., Brown J. M. (1997). Adaptation of human tumor cells to tirapazamine under aerobic conditions: implications of increased antioxidant enzyme activity to mechanism of aerobic cytotoxicity.. Biochem Pharmacol.

[PDF_00548] Evans J. W., Yudoh K., Delahoussaye Y. M., Brown J. M. (1998). Tirapazamine is metabolized to its DNA-damaging radical by intranuclear enzymes.. Cancer Res.

[PDF_00551] Forkert P. G., Lord J. A., Parkinson A. (1996). Alterations in expression of CYP1A1 and NADPH-cytochrome P450 reductase during lung tumor development in SWR/J mice.. Carcinogenesis.

[PDF_00554] Forrester L. M., Hayes J. D., Millis R., Barnes D., Harris A. L., Schlager J. J., Powis G., Wolf C. R. (1990). Expression of glutathione S-transferases and cytochrome P450 in normal and tumor breast tissue.. Carcinogenesis.

[PDF_00557] Jain R. K. (1988). Determinants of tumor blood flow: a review.. Cancer Res.

[PDF_00558] Laderoute K., Wardman P., Rauth A. M. (1988). Molecular mechanisms for the hypoxia-dependent activation of 3-amino-1,2,4-benzotriazine-1,4-dioxide (SR 4233).. Biochem Pharmacol.

[PDF_00565] Lloyd R. V., Duling D. R., Rumyantseva G. V., Mason R. P., Bridson P. K. (1991). Microsomal reduction of 3-amino-1,2,4-benzotriazine 1,4-dioxide to a free radical.. Mol Pharmacol.

[PDF_00574] Patterson A. V., Barham H. M., Chinje E. C., Adams G. E., Harris A. L., Stratford I. J. (1995). Importance of P450 reductase activity in determining sensitivity of breast tumour cells to the bioreductive drug, tirapazamine (SR 4233).. Br J Cancer.

[PDF_00570] Patterson A. V., Robertson N., Houlbrook S., Stephens M. A., Adams G. E., Harris A. L., Stratford I. J., Carmichael J. (1994). The role of DT-diaphorase in determining the sensitivity of human tumor cells to tirapazamine (SR 4233).. Int J Radiat Oncol Biol Phys.

[PDF_00582] Patterson A. V., Saunders M. P., Chinje E. C., Patterson L. H., Stratford I. J. (1998). Enzymology of tirapazamine metabolism: a review.. Anticancer Drug Des.

[PDF_00578] Patterson A. V., Saunders M. P., Chinje E. C., Talbot D. C., Harris A. L., Strafford I. J. (1997). Overexpression of human NADPH:cytochrome c (P450) reductase confers enhanced sensitivity to both tirapazamine (SR 4233) and RSU 1069.. Br J Cancer.

[PDF_00585] Plumb J. A., Workman P. (1994). Unusually marked hypoxic sensitization to indoloquinone EO9 and mitomycin C in a human colon-tumour cell line that lacks DT-diaphorase activity.. Int J Cancer.

[PDF_00588] Riley R. J., Hemingway S. A., Graham M. A., Workman P. (1993). Initial characterization of the major mouse cytochrome P450 enzymes involved in the reductive metabolism of the hypoxic cytotoxin 3-amino-1,2,4-benzotriazine-1,4-di-N-oxide (tirapazamine, SR 4233, WIN 59075).. Biochem Pharmacol.

[PDF_00592] Robertson N., Haigh A., Adams G. E., Stratford I. J. (1994). Factors affecting sensitivity to EO9 in rodent and human tumour cells in vitro: DT-diaphorase activity and hypoxia.. Eur J Cancer.

[PDF_00604] Workman P., Stratford I. J. (1993). The experimental development of bioreductive drugs and their role in cancer therapy.. Cancer Metastasis Rev.

